# Rapidly progressed neuroendocrine carcinoma in the extrahepatic bile duct: a case report and review of the literature

**DOI:** 10.1186/s40792-020-00945-3

**Published:** 2020-08-03

**Authors:** Mariko Kamiya, Naoto Yamamoto, Yuto Kamioka, Hirohide Inoue, Hirokazu Yotsumoto, Masaaki Murakawa, Toru Aoyama, Kota Washimi, Kae Kawachi, Takashi Oshima, Makoto Ueno, Norio Yukawa, Yasushi Rino, Munetaka Masuda, Soichiro Morinaga

**Affiliations:** 1 Department of Hepatobiliary and Pancreatic Surgery, Kanagawa Cancer Center, 1-1-2 Nakao, Asahi-Ku, Yokohama, 241-8515 Japan; 2 Department of Surgery, Yokohama City University, Yokohama, Japan; 3 Department of Pathology, Kanagawa Cancer Center, Yokohama, Japan; 4 Department of Gastrointestinal Surgery, Kanagawa Cancer Center, Yokohama, Japan; 5 Department of Gastroenterology, Hepatobiliary and Pancreatic Medical Oncology Division, Kanagawa Cancer Center, Yokohama, Japan

**Keywords:** Neuroendocrine carcinoma, Extrahepatic bile duct, Adenosquamous carcinoma

## Abstract

**Background:**

Neuroendocrine carcinoma (NEC) originating from the extrahepatic bile duct (EHBD) is very rare but is known for its aggressiveness and poor prognosis. We herein report a case of rapidly progressed NEC in the extrahepatic bile duct.

**Case presentation:**

An 84-year-old man was referred to our facility with obstructive jaundice and abdominal pain. Imaging studies revealed an irregular filling defect in the middle bile duct by endoscopic retrograde cholangiopancreatography and an enhanced wall thickening from the middle to distal portion by enhanced computed tomography. The patient was initially diagnosed with extrahepatic cholangiocarcinoma by a bile duct biopsy and underwent pancreatoduodenectomy with lymph node dissection. The pathological findings showed an NEC with an adenosquamous carcinoma component in the extrahepatic bile duct with lymph node metastases. The patient experienced multiple liver metastases 1 month after surgery and died 3 months after surgery. Due to the rapid progression of his disease, his general condition deteriorated, and he was unable to receive any additional treatments, such as chemotherapy.

**Conclusion:**

As shown in our case, NEC of the EHBD has an extremely poor prognosis and can sometimes progress rapidly. Multimodality treatment should be considered, even in cases of locoregional disease.

## Background

Neuroendocrine neoplasms (NENs) can arise in various organs through the body, but those arising in the gastrointestinal tract and the pancreas are relatively rare, accounting for 1–1.5% of all gastroenteropancreatic (GEP) neoplasms [1]. The annual age-adjusted incidence of GEP NENs in the USA was 3.56 per 100,000 persons in 2012, which is rare but steadily increasing [2]. The most common primary site of digestive system was the small intestine (1.05 per 100,000 persons), followed by the rectum (1.04 per 100,000 persons) and the pancreas (0.84 per 100,000 persons) [2]. Only 0.32% of NENs occur in the extrahepatic bile duct (EHBD), and almost all of them are well-differentiated neuroendocrine tumors (NETs) [3]. Poorly differentiated neuroendocrine carcinomas (NECs) of the EHBD are rare, reportedly accounting for only 0.19% of EHBD malignancies [4].

GEP NECs are an invasive and progressive disease for which the prognosis is extremely poor due to early widespread metastases [5, 6]. In the WHO 2019 classification, NENs of digestive system are classified into NETs and NECs according to their clinical and molecular differences [7]. The lesions previously classified as NET G3 (NEC) in the 2010 WHO classification were divided into NET G3 (well-differentiated high-grade tumor) and NEC (poorly differentiated high-grade tumor) in the 2019 classification system. Well-differentiated NETs have mutations in MEN1, DAXX, and ATRX. NECs are usually associated with TP53 or RB1 mutations, but NET G3 is not. These molecular differences underlie why progression from NETs to NECs does not generally occur and explain the differing clinical behavior of these two categories [7]. Based on the genomic data, the classification of mixed adenoneuroendocrine carcinomas (MANECs) was shifted to the conceptual category of “mixed neuroendocrine non-neuroendocrine neoplasms (MiNENs)” in the 2017 WHO classification system [7, 8]. These mixed neoplasms of digestive system are thought to have a common precursor, such as cancer stem cells that can differentiate into various cell lines [9].

EHBD NEC is also known for being difficult to diagnose preoperatively [3]. Many reported cases were resected with a diagnosis of cholangiocarcinoma and then diagnosed as NEC after surgery. We herein report a rare case of NEC in the EHBD that rapidly progressed after curative surgery and provide a brief review of the literature to further our understanding of this extremely rare and lethal malignancy.

## Case presentation

**Fig. 1:**
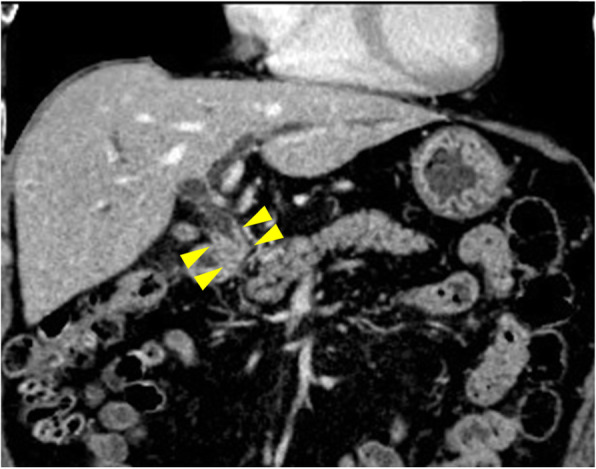
Enhanced wall thickening from the middle to the distal portion of the common bile duct (arrow) without any enlarged regional lymph nodes was detected by enhanced CT (coronal section image)

**Fig. 2:**
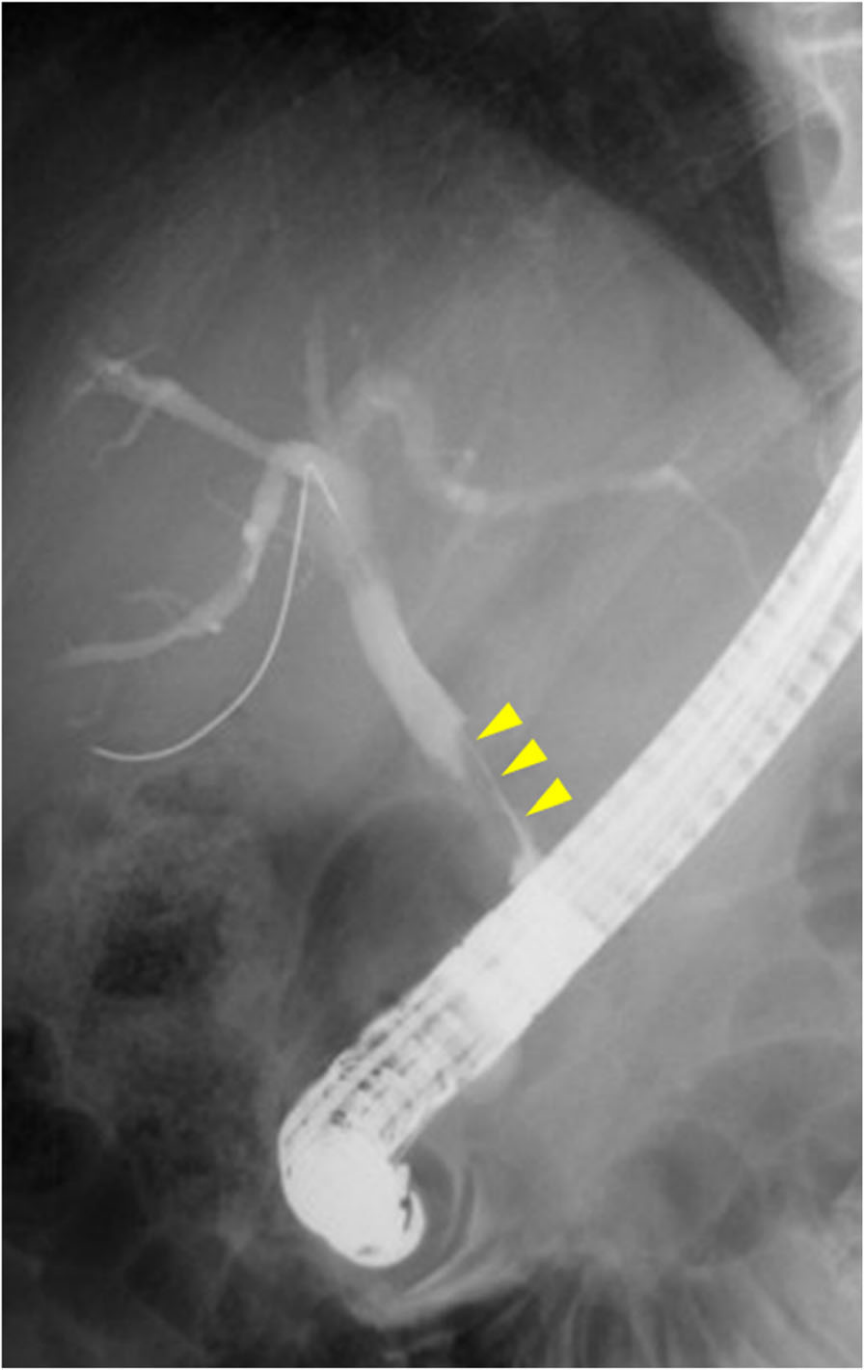
Endoscopic retrograde cholangiopancreatography revealed mild dilatation of the intra- and extrahepatic bile duct and an irregular filling defect in the middle portion of the common bile duct (arrow)

An 84-year-old man was referred to our hospital for the evaluation of obstructive jaundice and abdominal pain. He had no relevant medical history. On a physical examination, the patient presented with mild jaundice, itching of the skin, and mild discomfort in the upper abdomen. Laboratory tests revealed an elevated level of hepatobiliary enzyme and C-reactive protein (1.1 mg/dL). The serum level of carcinoembryonic antigen (CEA) was abnormally elevated (31.8 ng/mL), and the carbohydrate antigen 19-9 (CA19-9) level was within the normal range (8.2 U/mL). Enhanced computed tomography (CT) showed enhanced wall thickening from the middle to the distal portion of the common bile duct and no enlarged regional lymph nodes (Fig. 1). Endoscopic retrograde cholangiopancreatography (ERCP) demonstrated mild dilatation of the EHBD and an irregular filling defect in the middle bile duct (Fig. 2). Endoscopic ultrasonography revealed irregular wall thickening in the middle bile duct. A plastic stent tube was placed in the EHBD to reduce obstructive jaundice. We diagnosed him with extrahepatic cholangiocarcinoma because carcinoma was detected by a bile duct biopsy.

**Fig. 3:**
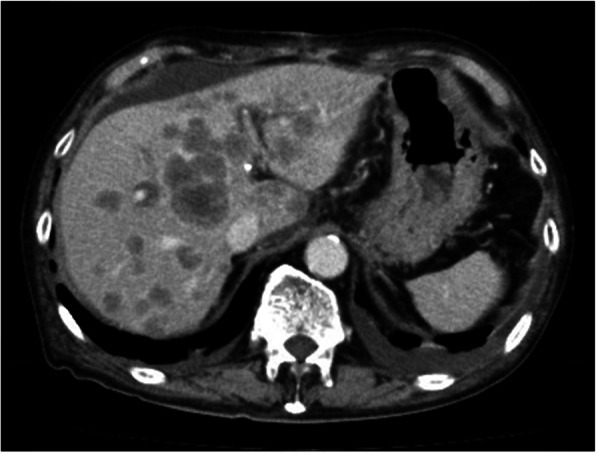
Multiple liver metastases and ascites on the liver surface were observed by CT (axial section image) 1 month after surgery

Pancreatoduodenectomy with lymph node dissection was performed. Heart failure and pancreatic fistula within Clavien-Dindo grade 3 occurred during the postoperative course, but those complications were improved after a few days, and he was discharged on postoperative day 23. One month after the surgery, the serum level of CEA was markedly elevated (306.4 ng/mL), and multiple liver metastases were detected by CT (Fig. 3). Due to the rapid progression of the disease, his general condition deteriorated, and he was unable to receive any additional treatments except for best supportive care. He deceased 3 months after the surgery.

### Pathological findings

**Fig. 4:**
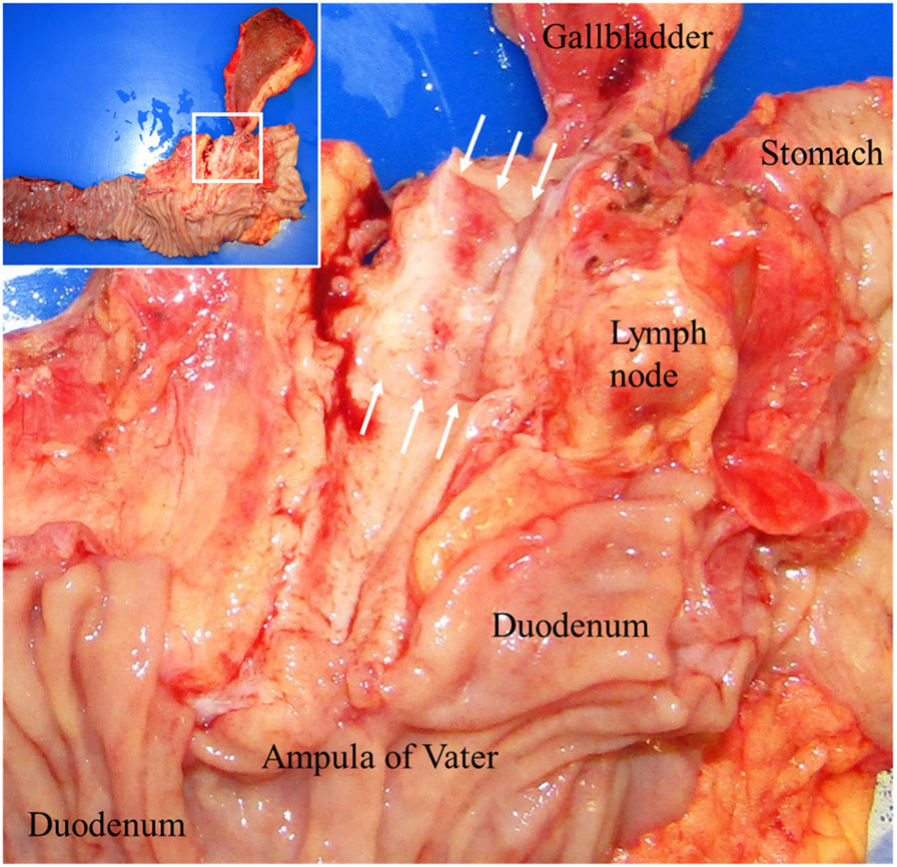
Macroscopic findings of the resected specimen showed a circumferentially flat, infiltrating lesion in the middle portion of the common bile duct around the junction of the cystic duct (arrow), measuring 25 × 23 × 10 mm in size

**Fig. 5:**
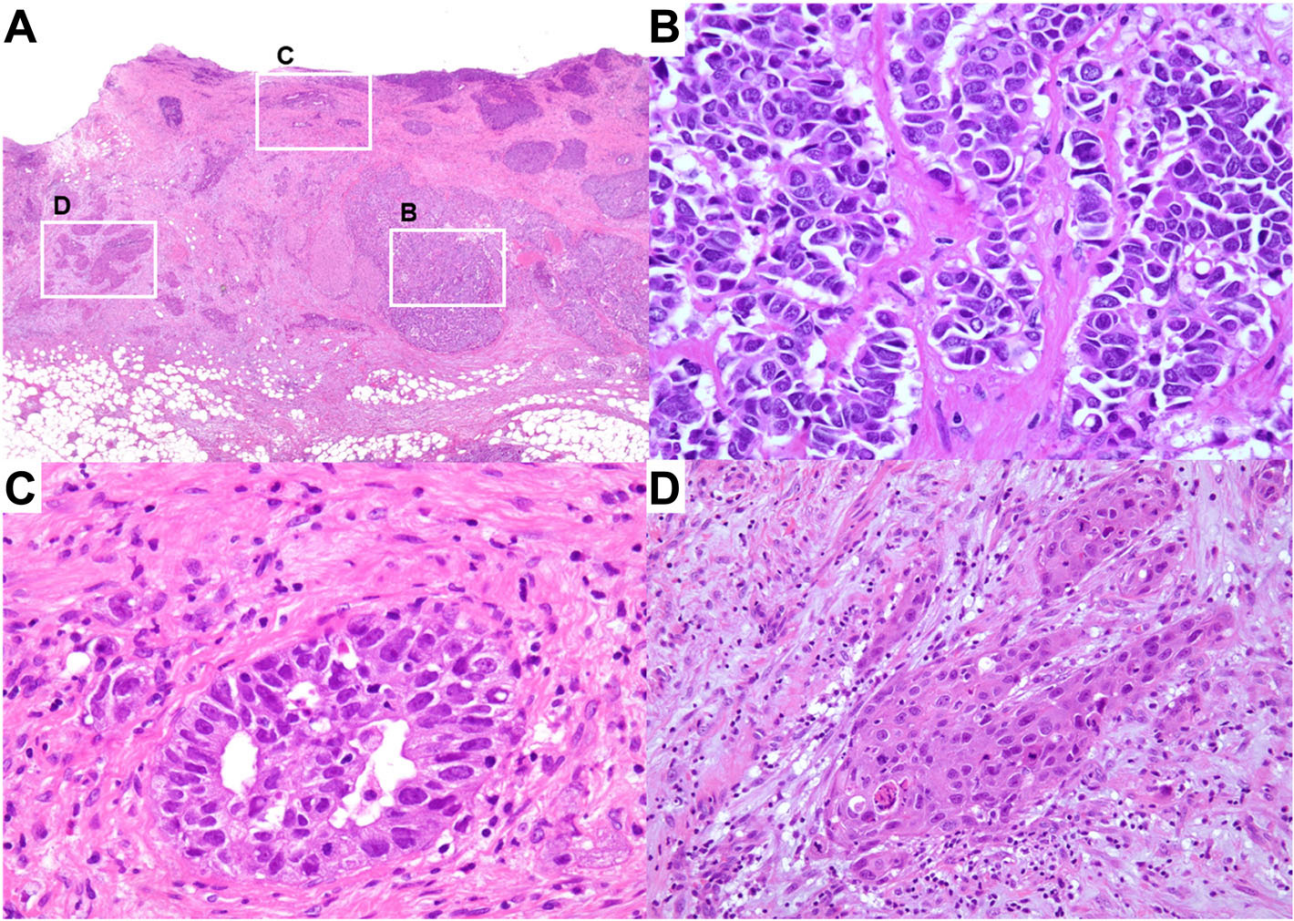
Microscopic findings of the resected specimen with Hematoxylin-Eosin (HE) staining. **a** An NEC lesion was observed below the submucosal layer (**b**). An adenocarcinoma lesion was detected in the surface layer (**c**). The tumor partly showed squamous cell carcinoma (**d**). **b** NEC cells were dysplastic cells with a high N/C ratio and granular hyperchromatic irregular-shaped nuclei. **c** Adenocarcinoma lesion. **d** Squamous cell carcinoma lesion

Macroscopically, the tumor was circumferentially a flat infiltrating lesion, measuring 25 × 23 × 10 mm located in the middle bile duct around the junction of the cystic duct (Fig. 4). Microscopically, the tumor cells were dysplastic cells with a high nucleo-cytoplasmic ratio (N/C ratio) and had granular hyperchromatic irregular-shaped nuclei. The tumor grew invasively, forming follicular nests and sheets (Fig. 5b). On immunostaining, the tumor cells were positive for synaptophysin, chromogranin A, and CD 56. The Ki-67 labeling index was over 80%. NEC was indicated based on these findings. In addition, the tumor partly showed adenocarcinoma (Fig. 5c) and squamous cell carcinoma areas (Fig. 5d). An adenocarcinoma region was found on the surface of the mucosa, extending and infiltrating into the cystic duct (Fig. 5a). The NEC region was mainly observed below the submucosal layer (Fig. 5a). The NEC component occupied over 80% of the tumor. Based on these findings, a pathological diagnosis of NEC with adenosquamous carcinoma components was established. Two regional lymph node metastases with NEC were detected (Bd-p, pT2, ly1, v3, ne2, pN1, M0, pStage IIB, UICC8th).

## Discussion

We reported an extremely rare case of NEC in the EHBD that was initially diagnosed as EHBD cancer by a bile duct biopsy and treated with curative resection but showed a rapid disease course after surgery and a poor prognosis. The optimum treatment for EHBD NEC has not been established due to its rarity. A detailed literary analysis of the clinical features of EHBD NECs may help improve the treatment of this lesion.

We searched the PubMed database using the terms “neuroendocrine carcinoma,” “bile duct,” “endocrine carcinoma,” and “mixed adeno-neuroendocrine carcinoma.” Excluding NECs in the intrahepatic bile duct, gallbladder, and ampulla of Vater, only 24 resected cases of primary EHBD NECs were found [3, 10–32]. We briefly summarized these clinical and pathological features (Tables 1 and 2).

**Table 1:** 25 reported cases of primary extrahepatic bile duct neuroendocrine carcinoma

Year	Author	Age/Sex	Location	Symptom	Preoperative diagnosis	Operation	Histology	Size (mm)	Ki 67 Index (%)	LN metastasis	Adjuvant therapy	Recurrence	RFS (months)	Recurrent organ	Outcome
2003	Kuraoka	75M	Bd	jaundice	BDC	PD	SCNEC	45	90	+	ND	+	5	LN	ND
2003	Hazama	60M	Bd	jaundice	SCC	PD	SCNEC	3	ND	+	-	+	11	brain, LN	12M dead
2005	Kaiho	66F	Bd	pain	BDC	PD	SCNEC + a	35	ND	ND	+, ND	+	ND	liver	8M dead
2006	Sato	68M	Bd	jaundice	BDC	PD	LCNEC + a	20	71.4	-	ND	+	2	liver	3M dead
2009	Kohashi	77M	Bd	jaundice	BDC	PD	LCNEC	18	67	-	-	+	3	liver, lung, local	3M dead
2009	Okamura	62M	Bd	abdominal pain, fever	SCC	PD	SCC	30	ND	ND	+, IP	+	8	bone	20M dead
2011	Masui	82M	Bd	jaundice, anorexia	BDC	EHBDR	AECC	25	35	-	-	+	3	liver	6M dead
2012	Takahashi	28F	Bd	pruritus	BDC	PD	NEC	30	89.8	-	-	-	36	none	36M alive
2013	Sasatomi	76M	Bp	jaundice	BDC	H + EHBDR	LCNEC	50	75	+	-	+	0	LN	21days dead
2013	Linder	82M	Bd	jaundice, abdominal pain, weight loss	BDC	PD	MANEC	19	ND	+	-	-	6	none	6M alive
2014	Lee	75M	Bd	jaundice	BDC	EHBDR	MANEC	20	ND	-	-	-	11	none	11M alive
2014	Wysocki	65M	Bp	jaundice, vomit, weight loss	ND	EHBDR	LCNEC + a	36	80	ND	-	ND	ND	ND	5M dead
2014	Park SB	75F	Bd	nausea, jaundice	BDC	EHBDR	LCNEC	27	ND	+	+, 5-FU+CDDP	+	7	liver, local	12M dead
2015	Aigner	61M	Bd	abdominal pain, icterus, nausea, pruritus	SCNEC	EHBDR	SCNEC	27	90	ND	ND	+	3	liver, LN, bone	ND
2015	Kihara	70F	Bp	jaundice	BDC	H + EHBDR	SCNEC	30	70	+	+, CPT-11+CBDCA	-	10	none	10M alive
2016	Priyanka	76M	Bp	jaundice, weight loss	BDC	EHBDR	MANEC	14	90	-	-	ND	ND	ND	ND
2016	Murakami	80M	Bp	jaundice	BDC	EHBDR	LCNEC + a	24	72	+	-	+	2.5	Liver, lung, P	3M dead
2016	Oshiro	72M	Bp	jaundice	BDC	H + EHBDR	LCNEC	30	56.2	+	-	+	3	Liver	7M alive
2017	Izumo	66M	Bd	jaundice, anorexia, fatigue	BDC	PD	MANEC	10	30	+	-	-	30	none	30M alive
2017	Komo	82M	Bd	Liver dysfunction	BDC	PD	MANEC	18	37	-	-	-	7	none	7M alive
2018	Zhang L	62M	Bp	jaundice	BDC	H + EHBDR	NEC	20	80	+	-	+	2	liver	6M dead
2018	Park JY	59M	Bp	jaundice	BDC	EHBDR	LCNEC	62	20	-	+, RT+VP-16+CDDP	-	10	none	10M alive
2019	Zhang HW	60M	Bp	abdominal pain	ND	EHBDR	MANEC	17	70	+	ND	ND	ND	ND	ND
2019	Zhang L	64F	Bd	jaundice, abdominal pain	BDC	PD	MANEC	45	50	ND	-	+	5	liver, lung	12M dead
2020	our case	84M	Bd	jaundice	BDC	PD	SCNEC + a	25	80	+	-	+	1	liver	3M dead

*LN* lymph node, *RFS* recurrence-free survival, *Bd* distal bile duct, *Bp* perihilar bile duct, *BDC* bile duct cancer, *SCC* small-cell carcinoma, *ND* no data, *SCNEC* small-cell neuroendocrine carcinoma, *PD* pancreatoduodenectomy, *EHBDR* extrahepatic bile duct resection, *H+* hemihepatectomy+, *+a* +adenocarcinoma, *LCNEC* large-cell neuroendocrine carcinoma, *AECC* adenoendocrine carcinoma, *MANEC* mixed adenoneuroendocrine carcinoma, *IP* irinotecan+cisplatin, *5-FU* 5-fluorouracil, *CDDP* cisplatin, *CPT-11* irinotecan, *CBDCA* carboplatin, *RT* radiotherapy, *VP-16* etoposide, *P* peritoneal metastasis

**Table 2:** Summary of 25 reported cases of primary extrahepatic bile duct neuroendocrine carcinoma

**Sex** (*n*=25)	Male	20	**Operation** (*n* =25)		
	Female	5		Pancreatoduodenectomy	12
**Age** (*n*=25)	median 70 years (range 28-84)			Bile duct resection	9
**Symptom**	Jaundice	22		With hepatectomy	4
	Abdominal pain	6	**Lymph node metastasis** (*n*=20)		
	Weight loss	3		Positive	12
	Nausea, Vomiting	3		Negative	8
**Preoperative diagnosis** (*n*=25)			**Adjuvant chemotherapy** (*n*=20)		
	Bile duct cancer	20		Yes	5
	Neuroendocrine carcinoma	3		No	15
	Not mentioned	2	**Neoadjuvant chemotherapy**		3
**Location** (*n*=25)	Bd	16	**Recurrent organ** (*n*=15)		
	Bp	9		Liver	11
**Size** (*n*=25)	median 25mm (range 3-62)			Lymph node	4
**Ki-67 index** (*n*=19)				Lung	3
	median 71.4% (range 20-90)			Local / Bone	2

Among the 25 resected cases of EHBD NEC (including our case), the median age of the patients was 70.0 years old (range 28–84). Most patients were male, with a male to female ratio of 20:5. Most of patients showed primary symptoms of obstructive jaundice (22 cases), followed by abdominal pain (6 cases), weight loss (3 cases), and nausea (3 cases), similar to EHBD cancer. The tumor was located in the perihilar bile duct in 9 cases and in the distal bile duct in 16 cases. The serum levels of CEA and CA 19-9 were abnormally elevated in some cases, regardless of the presence of adenocarcinoma component, but these are not specific tumor markers for biliary NECs. The median Ki-67 index was 71.4% (range 20–90%, *n* = 19). Pancreatoduodenectomy was performed in 12 cases, EHBD resection in 9 cases, and hemihepatectomy with EHBD resection in 4 cases. Lymph node metastases were detected in 12 of 20 cases (Table 2).

It is very difficult to make a diagnosis of NEC preoperatively. The clinical and imaging findings of EHBD NEC are very similar to that of EHBD cancer, so a histological examination is required for a definitive diagnosis. A bile duct biopsy was performed in 10 cases, only 3 of whom were diagnosed with NEC before resection [11, 15, 23]. Adenocarcinoma was detected in four cases, and atypical cells or no malignant cells were detected in three cases. Brushing cytology was performed in 11 cases, but NEC could not be detected in any of these cases (adenocarcinoma in 4 cases, atypical cells or no malignant cells in 7 cases). Consequently, 20 patients underwent surgery with a diagnosis of EHBD cancer. The preoperative diagnosis was not mentioned in two cases (Table 2).

One reason for the difficulty associated with making a preoperative pathological diagnosis is that a relatively high proportion of NEC cases have an adenocarcinoma component (52%, 13 of 25 cases), and this adenocarcinoma component in the superficial layer conceals the NEC component existing in a deeper layer. Approximately 35% of biliary NENs are MiNENs, and many cases often contain non-neuroendocrine components, as in our case, even if they do not meet the definition of MiNEN (both neuroendocrine and non-neuroendocrine components exceed 30%) [8]. According to Sasatomi et al., in cases of bile duct NEC with an adenocarcinoma component, the adenocarcinoma lesion is often found in the mucosal to submucosal layer, whereas the NEC lesion is found below the submucosal layer or in an even deeper layer [18]. Therefore, in some cases, cytology or a biopsy cannot detect the NEC component, making a preoperative diagnosis difficult.

Another possible reason is that cytology specimens stained with Papanicolaou and biopsy specimens stained with Hematoxylin-Eosin (HE) alone may have a high false negative rate for diagnosing NEC [3]. Immunohistochemical staining is usually required for a definitive diagnosis of NEC, but pathologists do not always conduct this unless NEC is suspected. In our case, the patient was diagnosed with bile duct cancer based on HE staining alone preoperatively, and NEC was diagnosed postoperatively.

**Fig. 6:**
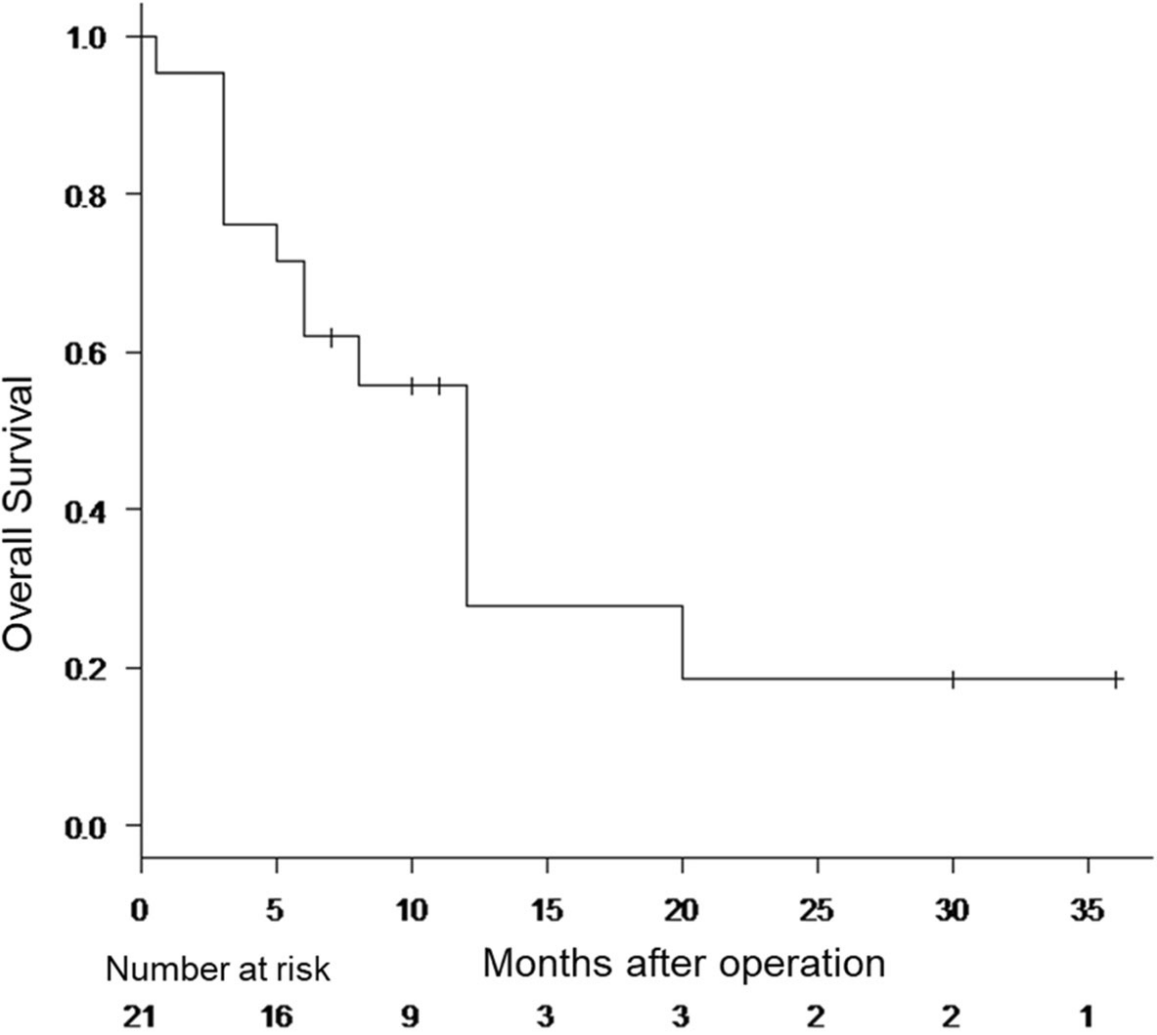
Survival curves of the 21 resected cases of EHBD NEC. The median OS was 12 months (95% CI, 5–20 months), and the 1-year survival rate was 27.9%

The prognosis of EHBD NECs is very poor, even in cases of lesions that are clinically localized and surgically resected. The median overall survival was 12 months (95% confidence interval, 5–20 months) in the 21 cases with follow-up data (Fig. 6). There was no significant difference in the overall survival between patients with and without an adenocarcinoma component in the present study, although the overall survival of biliary MiNEN is reported to be slightly better than that of pure NEC in some literature. However, advanced MiNEN generally shows a relatively poor prognosis that is equal to that of pure NEC [8]. Only 2 patients were reported to survive for more than 2 years. One case was pStage I (T1N0M0) with a tumor size of 30 mm and remained alive for 36 months, while the other case was pStage IIB (T3N1M0) with a tumor size of 10 mm and remained alive for 30 months. No recurrence was observed in either case without any adjuvant therapies. Postoperative recurrence occurred in 15 cases, and the most common recurrent organ was the liver (*n* = 11), followed by the lymph nodes (*n* = 4), and the lung (*n* = 3).

For the treatment of GEP NENs, resection is recommended for well-differentiated NETs (including NET G3) that can be radically resected. However, the role of surgery for GEP NECs is limited, as GEP NEC has a rapid disease course with a poor prognosis that can relapse early after resection, even in cases of clinically localized tumors. Generally, the combination of systemic chemotherapy (neoadjuvant or adjuvant) and local treatment consisting of surgery and radiotherapy should be considered for localized GEP NEC [5]. GEP NECs are chemotherapy-responsive neoplasms, and platinum-based chemotherapy represents the backbone of treatment for both early and advanced-stage GEP-NEC [6].

In the literature, adjuvant chemotherapy, mostly based on regimens for small-cell lung carcinoma (SCLC), was performed in only 5 cases (*n* = 21). Due to the rapid disease course after surgery, a certain number of patients with resected EHBD NEC were unable to receive adjuvant chemotherapy, which was the same as in our case. Notably, the three cases diagnosed with NEC preoperatively all received neoadjuvant chemotherapy with IP (irinotecan + cisplatin) or EP (etoposide + cisplatin) [11, 15, 23]. Hazama et al. reported a case of unresectable EHBD NEC due to para-aortic lymph node metastasis that was resected after four cycles of EP. A partial response was obtained, and the residual tumor was only 3 mm in size with a single lymph node showing one tiny metastasis [11].

Neoadjuvant chemotherapy has some advantages over adjuvant chemotherapy [15, 33]. Many NEC patients already have occult metastases at the time of their diagnosis, resulting in early recurrence and progression after surgery. Neoadjuvant chemotherapy with a platinum-based regimen can control these occult metastases due to its relatively good tumor sensitivity [6, 34]. Furthermore, before surgery, patients can maintain good activities of daily living and a good general condition, which allows for more aggressive treatment to be administered. From this perspective, neoadjuvant treatment might be preferred to adjuvant treatment for patients with EHBD NEC, even cases with clinically localized and surgically resectable lesions.

## Conclusion

In summary, we reported a case of resected EHBD NEC with a rapid disease course and poor prognosis. The preoperative diagnosis of primary EHBD NEC is very difficult, and this lesion has an extremely poor prognosis and can progress rapidly after surgery. Multimodality treatment including chemotherapy (neoadjuvant rather than adjuvant), radiotherapy, and surgery should be carefully considered to prolong the survival of patients with EHBD NEC.

## Data Availability

The dataset supporting the conclusions of this article is included within the article and its additional files.

## Abbreviations

NEN: Neuroendocrine neoplasm
GEP: Gastroenteropancreatic
EHBD: Extrahepatic bile duct
NET: Neuroendocrine tumor
NEC: Neuroendocrine carcinoma
MiNEN: Mixed neuroendocrine non-neuroendocrine neoplasm
CT: Computed tomography
ERCP: Endoscopic retrograde cholangiopancreatography
SCLC: Small-cell lung carcinoma
IP: Irinotecan + cisplatin
EP: Etoposide + cisplatin
